# Body size modulates the extent of seasonal diet switching by large mammalian herbivores in Yellowstone National Park

**DOI:** 10.1098/rsos.240136

**Published:** 2024-09-11

**Authors:** Bethan L. Littleford-Colquhoun, Chris Geremia, Lauren M. McGarvey, Jerod A. Merkle, Hannah K. Hoff, Heidi Anderson, Carlisle R. Segal, Rebecca Y. Kartzinel, Ian J. Maywar, Natalie Nantais, Camela Moore, Tyler R. Kartzinel

**Affiliations:** ^1^ Department of Ecology, Evolution & Organismal Biology, Brown University, Providence, RI 02912, USA; ^2^ Institute at Brown for Environment and Society, Brown University, Providence, RI 02912, USA; ^3^ Yellowstone Center for Resources, Yellowstone National Park, Mammoth Hot Springs, Mammoth, WY 82190, USA; ^4^ Department of Zoology and Physiology, University of Wyoming, Laramie, WY 82071, USA; ^5^ Yellowstone Herbarium, Yellowstone National Park, Mammoth Hot Springs, Mammoth, WY 82190, USA; ^6^ Brown University Herbarium, Brown University, Providence, RI 02912, USA; ^7^ Southern Illinois University, Carbondale, IL 62901, USA

**Keywords:** coexistence, food web ecology, specialization, niche variation hypothesis, optimal foraging theory, resource limitation

## Abstract

Prevailing theories about animal foraging behaviours and the food webs they occupy offer divergent predictions about whether seasonally limited food availability promotes dietary diversification or specialization. Emphasis on how animals compete for food predominates in work on the foraging ecology of large mammalian herbivores, whereas emphasis on how the diversity of available foods generally constrains dietary opportunity predominates work on entire food webs. Reconciling predictions about what promotes dietary diversification is challenging because species’ different body sizes and mobilities modulate how they seek and compete for resources—the mechanistic bases of common predictions may not pertain to all species equally. We evaluated predictions about five large-herbivore species that differ in body size and mobility in Yellowstone National Park using GPS tracking and dietary DNA. The data illuminated remarkably strong and significant correlations between body size and five key indicators of diet seasonality (*R* ^2^ = 0.71–0.80). Compared to smaller species, bison and elk showed muted diet seasonality and maintained access to more unique foods when winter conditions constrained food availability. Evidence from GPS collars revealed size-based differences in species’ seasonal movements and habitat-use patterns, suggesting that better accounting for the allometry of foraging behaviours may help reconcile disparate ideas about the ecological drivers of seasonal diet switching.

## 1. Introduction

A cornerstone of ecology involves elucidating the diversity of species that an environment can support, which is limited by the availability of resources and the degree to which species specialize on distinct resources through space and time [1,2]. Two prominent theoretical frameworks provide predictions about how animals respond to changing resource availability: one focuses on animals’ foraging behaviours [3–6] and the other focuses on the emergent properties of complex food webs in which these animals occur [7]. Ecologists increasingly recognize that these frameworks lead to divergent predictions even though they attempt to solve similar ecological problems. This raises the question of whether these two bodies of theory present alternative hypotheses or if they are perhaps more complementary and contingent on the functional characteristics of consumers in the community. Well-established bodies of literature that surround both animal foraging behaviours and community dynamics emphasize how seasonal variation in resource availability and consumer body size intersect to modulate the diversity of foods that animals may eat [8–10]. Variation in animal foraging behaviour inevitably scales up to affect the topology of the complex food webs in which these animals reside.

**Figure 1.**
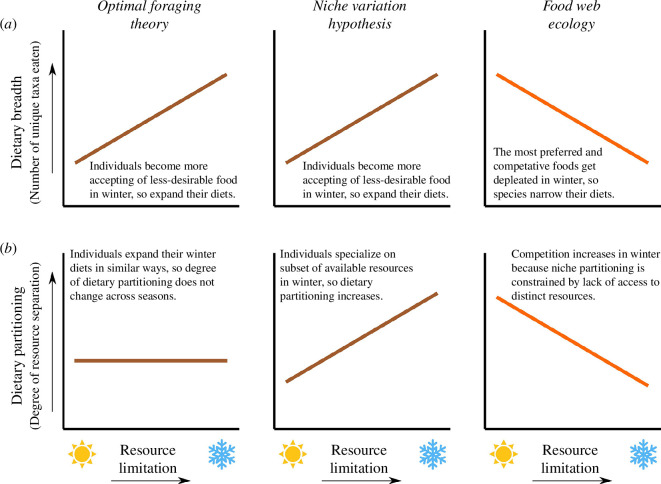
Three sets of predictions about how large mammalian herbivores should respond to seasonal resource variation in terms of (*a*) dietary breadth and (*b*) dietary partitioning. The combinations of predicted responses differ according to foraging ecology (brown lines; optimal foraging theory and niche variation hypothesis) versus food web ecology (orange lines).

The field of foraging ecology focuses on how animals navigate their landscape to find adequate forage while meeting all other requirements [11]. Depending on the species’ biology, some populations forage as a broad group while members of others may forage more individualistically [3,8,12]. Accordingly, within foraging ecology, two hypotheses aim to account for the types of foraging decisions that consumers should make—optimal foraging theory (OFT) and the niche variation hypothesis (NVH)—and share some key predictions. Both hypotheses predict that when food becomes limited and competition increases, animals must become accepting of less-desirable options and thus dietary diversity should increase (figure 1*a*) [4]. Yet a classical assumption of OFT is that members of the same species share food preferences and thus tend to make similar decisions about what to eat [6,13], resulting in little dietary partitioning among individuals, regardless of resource availability (figure 1*b*). Recent technological advances in GPS tracking and fine-grained dietary assessments have, however, revealed individual variations in behaviour [14], morphology [15] and physiology [9,16] that can promote individualistic foraging strategies. Consequently, NVH emphasizes how these variable attributes of consumers can promote independent foraging decisions, which can ameliorate intra-specific competition as members of the same population use different subsets of available foods, especially during times of resource limitation (figure 1*b*) [3,5,8,17]. Consumer foraging behaviours can have profound effects on the long-term productivity and diversity of food webs [18], but how intra-specific variation in these foraging behaviours can be expected to generate differences in the structure and function of food webs is largely unexplored.

At higher scales of biological organization, food web ecology focuses on the emergent properties of feeding networks through space and time [10,19]. In contrast to foraging ecology, food web ecology predicts that when nutritious resources are abundant and diverse, consumers generally benefit from expanding their dietary diversity, but when those preferred resources become limited, they may be forced to converge on a smaller subset of overlapping resources, reducing dietary diversity and dietary partitioning (figure 1). Although food web research has long focused on these types of broad-scale variation in consumer–resource interactions, new high-resolution dietary data show that these networks can be plastic and contain more cryptic dietary partitioning than we previously thought [9,20,21]. According to a recent review of foraging theories [8], the benefits that populations derive from adopting unique diets are expected to be maximized when resources are most limited because this is when competition will be most intense both between species (per OFT) and also within them (per the NVH; figure 1*b*) [22,23]. Importantly, resource-limited conditions also impose the greatest constraints on dietary partitioning—if indeed it is even possible—because the lack of resources provided by the ecosystem could prevent individuals from pursuing alternatives (per food web ecology; figure 1*b*) [12,19]. Whether the maximum extent of diet partitioning among consumers is realized at times when food webs provide the most abundant and diverse resources versus times when access to resources are most constrained remains an open question.

One of the best-studied food webs in the world is that of the Greater Yellowstone Ecosystem. Yellowstone boasts the most diverse large-herbivore community in North America where drastic seasonal changes in resource availability and quality drive long-distance migrations of bison, elk, mule deer, bighorn sheep and pronghorn [24–28]. These migrations follow characteristic annual patterns: during winter, when resources are limited and of low quality, individuals of all species tend to occupy lower elevation habitats; in spring, they follow the ‘green wave’ of emergent plants to higher elevations; in summer, they take advantage of a greater diversity and abundance of high-quality forage at higher elevations; and in autumn, they migrate back towards their winter ranges [29,30]. Despite sharing these canonical migratory patterns, the ungulates of Yellowstone vary greatly in body size, movement patterns, social systems and habitat use—all of which are likely drivers of dietary differences [31]. Larger herbivores, such as bison, should generally be able to persist on larger quantities of low-quality/high-biomass foods at times when or in places where resources are limited [32,33]. In contrast, smaller herbivores, such as pronghorn, should generally prioritize foods of the highest quality [34–36]. In communities like these, body size differences between species may promote differences in foraging behaviours that in turn modulate the degree to which habitat- and food-use strategies diverge. Elucidating relationships between body size and the extent to which animals seasonally switch resource-acquisition strategies could help reconcile divergent predictions about when the uniqueness of diet profiles should be maximized within and between species (figure 1) [31,36].

We developed an unparalleled community-level dataset based on five ungulate species from Yellowstone National Park that allowed us to evaluate divergent predictions across scales of biological organization from individual herds nested within species to species nested within the broader community. We used 2 years of GPS-tracking and dietary DNA-metabarcoding data to elucidate dietary variation across times of resource limitation and resource abundance. We hypothesized that the effect of seasonality would be modulated by body size for (i) the degree of intra-specific dietary switching, (ii) herd-level dietary richness, (iii) species-level total niche width (TNW), (iv) herd-level dietary uniqueness, and (v) species-level dietary uniqueness.

## 2. Methods

### 2.1. Study system

The Greater Yellowstone Ecosystem harbours the most diverse large-mammal assemblage in North America, and we focused on the five predominant members of the community. We obtained high-resolution diet profiles for pronghorn (*Antilocapra americana*; 48 kg adult body mass), bighorn sheep (*Ovis canadensis*; 75 kg), mule deer (*Odocoileus hemionus*; 85 kg), elk (*Cervus canadensis*; 241 kg) and bison (*Bison bison*; 625 kg). Average body size estimates for each species were obtained from panTHERIA [37]. With increasing elevation across Yellowstone, habitats dominated by grasses and shrubs are replaced by lodgepole pine forests and then subalpine forests of spruce and fir [38]. Pronounced seasonal changes in vegetation follow a cycle: rapid vegetation emergence occurs in spring (March–May), summers (June–August) are rich in plant diversity, plant senescence begins in early autumn (September–November), and winter (December–February) brings a snow-covered period when vegetation is scarce and difficult to access except along ridges and in valleys [39].

### 2.2. Animal tracking and dietary sampling

All animal tracking and sampling protocols were established by the National Park Service (Research permit ID: YELL 2021-078 and IMR_YELL_White_Ungulates_2022.A3). We fitted GPS collars to 6–57 females per species and repeatedly sampled their herds for dietary analysis by collecting fresh faecal samples that were no more than a few hours old. Collars were set to record GPS locations every 1–4 h depending on species and season (the collars of bison, mule deer and bighorn sheep had 2 h GPS fixes, pronghorn had 4 h GPS fixes due to battery limitations, and the GPS fixes for elk varied from 1 to 4 h throughout the year with shorter intervals typically in winter). We obtained dietary samples from herds that included GPS collared individuals from December 2016 to January 2018 (electronic supplementary material, dataset S1) and included samples collected across all four seasons. The National Park Service’s sampling strategy enabled participation by community science volunteers, concentrated in the northern sector of the park (72–95% of samples per species), though we also obtained samples from herds occupying Hayden Valley and the areas surrounding Yellowstone Lake to the south (electronic supplementary material, figure S1). Following traditional rangeland ecology sampling strategies [40,41], observers collected, combined and thoroughly mixed approximately equal volumes of fresh dung from 1 to 5 piles per herd (approx. 5 g per dung pile). Samples therefore represent dietary profiles for herds of each species. Since dietary DNA likely represents 24–72 h of foraging activity [42], we used the GPS data to quantify movement and habitat use by the collared individual over 72 h prior to sampling (yielding 6–81 fixes per individual; spatial data for six faecal samples could not be determined and therefore were not included in spatial analyses). A trajectory of the individual’s movement was created from the GPS fix locations, and a 400 m buffer was added to create a polygon representing 72 hours of locality data. Within each polygon, we quantified mean elevation (m), total area occupied (km^2^ polygon), coarse number of land cover types (i.e. habitat richness) and the proportion of each land cover types using datasets of 30 m resolution. Mean elevation was calculated using the US Geological Survey National Elevation Dataset, and land types covered by the polygon were identified using the National Land Cover Database (NLCD), a remotely sensed dataset of 16 different land cover classes from Landsat Thematic Mapper [43].

### 2.3. Dietary DNA metabarcoding

We extracted DNA from 371 faecal samples and amplified the chloroplast trnL-P6 marker using PCR [44] (electronic supplementary material, appendix S1). The trnL-P6 marker was chosen because of its relatively short length, conserved primer sites and taxonomic precision [21,44,45]. To obtain dietary profiles, we produced 2 × 150 bp paired-end Nextera libraries for sequencing on Illumina MiSeq (electronic supplementary material, appendix S1). To identify dietary DNA sequences, we developed two reference libraries (electronic supplementary material, appendix S2): the ‘local’ library comprised 191 unique trnL-P6 sequences from 416 specimens representing 45 plant families from Yellowstone (electronic supplementary material, dataset S2); the ‘global’ library was built using data from the European Molecular Biology Laboratory (release 143), which yielded 21 422 unique trnL-P6 sequences representing at least 615 plant families (electronic supplementary material, appendix S2). To produce diet profiles, we merged paired-end Illumina sequence reads and eliminated putative errors (electronic supplementary material, appendix S3). When inferring the taxonomy of dietary sequences to be included in the final diet profiles, we required a 100% match between each dietary sequence and a reference sequence from at least one of the libraries (electronic supplementary material, appendix S3; dataset S3). After removing one sample with <1000 sequence reads, we rarefied the data to equal read counts (*n* = 1453 reads per sample). The final dataset included 370 samples (winter = 90, spring = 153, summer = 93, autumn = 34; electronic supplementary material, table S1) and 685 plant taxa (94% identified to family, 65% to genus and 42% to species; electronic supplementary material, dataset S4). As food web ecology studies traditionally lump similar species into functional groups, we also assigned the plant taxa we identified genetically into functional groups (e.g. coniferous trees, non-coniferous trees, forbs, grasses, non-grass graminoids or shrubs; electronic supplementary material, appendix S3). We initially inferred plant functional types using the USDA Plants Database and we filled gaps in the USDA dataset with our knowledge of the local flora, providing functional classifications for 84% of the 685 plant taxa included in the final dataset (electronic supplementary material, appendix S3). We could not taxonomically assign the remaining 16% of DNA sequences to a functional group because currently available plant DNA reference data (i) provide incomplete precision in some taxonomic assignments and/or (ii) positively identified closely related species that vary in growth form; we thus included an ‘undetermined’ plant functional grouping in our analyses (electronic supplementary material, appendix S3). Plant functional type is not equivalent to taxonomic assignment and resulted in some superfamilies being split into different functional groups; the graminoids, for example, were split into the grasses (Poaceae) and non-grass graminoids (Cyperaceae, Juncaceae and Typhaceae).

### 2.4. Quantifying dietary variation

We first quantified overall dietary variation within and among species. At the herd level, we calculated dietary richness as the number of unique plant taxa and functional types (inclusive of the ‘undetermined’ functional group) per sample and quantified plant taxonomic and functional dissimilarity between herds using the Bray–Curtis metric [46] in vegan [47] in R [48]. Bray–Curtis dissimilarity is bounded between 0 (total similarity) and 1 (total dissimilarity). We tested for significant differences in diet composition according to herbivore species, season (summer, June–August versus winter, December–February) and the species × season interaction using adonis2 with 999 permutations. We also quantified multivariate dispersions (i.e. between-herd variance) of each species and season (summer versus winter) using betadisper with 999 permutations and bias adjustment. As multivariate dispersion calculates the distance between each sample and the centroid of its group (e.g. species or season), we compared the mean dispersion of groups using permutest.betadisper. Finally, we visualized dietary dissimilarity with non-metric multidimensional scaling (NMDS) using metaMDS; this was run with and without the inclusion of the ‘undetermined’ functional group. When visualizing dietary dissimilarity without the undetermined category, samples were re-rarefied once this group was removed.

We then quantified differences in intra-specific dietary richness, uniqueness, and switching between summer and winter. To account for variation in sample sizes across species and season (electronic supplementary material, table S1) and to enable sensitivity analyses related to our hypotheses about the allometry of diet switching, we based our analyses on 100 random subsamples of the dietary profiles representing each species–season combination [49,50]. From the 183 summer and winter diets (winter = 90, summer = 93; electronic supplementary material, table S1), we randomly drew the minimum number of dietary profiles in any species–season combination (*n* = 4; electronic supplementary material, table S1) and repeated this random draw 100 times. For each of these 100 subsamples, we calculated mean dietary richness and Bray–Curtis dissimilarity for all species–season combinations. We then used each of the 100 random subsamples to calculate the total number of plant taxa consumed (TNW) for each species–season combination using the incidence frequencies of plant taxa with the *estimateD* function in *iNEXT* v2.0.20 [51]. We compared TNW metrics based on this asymptotic estimator at the common value of *n* = 8 samples. Finally, to measure intra- and inter-specific dietary uniqueness in summer and winter, we calculated Blüthgen’s *d*′ specialization index [52]. The intuition behind *d*′ is that if a diet profile includes few plant taxa, but those taxa are common across many other diet profiles in the network, then it is not especially unique despite its narrow breadth. In contrast, a diet profile that includes many taxa that are rare across the network is relatively specialized. We calculated dietary uniqueness (*d*′) based on the 100 random subsamples using the *specieslevel* function in *bipartite* [53,54] and calculated *d*′ at (i) the herd level by calculating the uniqueness of each sample relative to other samples from the same season and taking the mean herd-level *d*′ values for each species and (ii) the species level by determining the mean diet profile of each species in each season and calculating the dietary uniqueness of each species’ mean diet profile relative to other species in the same season.

### 2.5. Hypothesis testing

To test our hypotheses about the allometry of species’ summer–winter diet transitions, we first tested for a significant correlation between body size and the degree of seasonal diet turnover (mean intra-specific Bray–Curtis dissimilarity) using linear regression, with log-transformed data on body size (g). Then, for each measure of dietary richness and specialization, we evaluated ANCOVAs with predictor variables that included body size, season and a body size × season interaction using Type III Sums of Squares. If we found homogeneity of regression slopes using likelihood-ratio tests, we eliminated the body size × season interaction and simplified to an additive model. We further simplified this additive model if likelihood-ratio tests indicated that removing one of the predictor variables (body size or season) did not yield a significantly worse model. We assessed the robustness of these main results by visualizing variation in regression slopes and intercepts based on the 100 random subsamples.

Finally, we evaluated associations between seasonal variation in diet, habitat use and movement patterns. We tested for significant correlations between dietary dissimilarity and habitat-use dissimilarity (Bray–Curtis dissimilarity of land-type proportion data) using Mantel tests with 999 permutations in *vegan*. We developed a visual heuristic to compare the effect of season on dietary switching (richness and Bray–Curtis), area occupied (based on GPS), and habitat-use richness (based on the NLCD) for each species. To quantify effect sizes for richness, area, and habitat use, we calculated mean log-response ratios (summer/winter).

## 3. Results

### 3.1. Space and habitat usage

**Figure 2.**
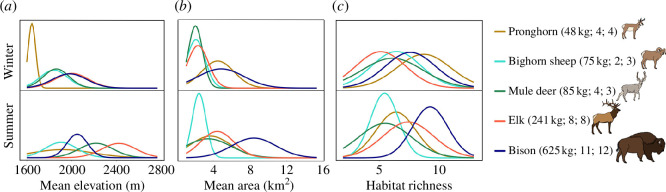
Movement and habitat use by animals in herds sampled for dietary DNA in Yellowstone National Park. Overall, samples from each species were obtained from (*a*) lower elevations (m), (*b*) smaller areas (km^2^) and (*c*) fewer habitat types (habitat richness) during winter. Collared animals were tracked by the National Park Service between December 2016 and January 2018, depending on species. The average body size of each species, the number of collared animals in winter and the number of collared animals in summer that were used to calculate movement data are shown in parentheses.

Patterns of space and habitat use differed between the herds we sampled in summer and winter. The GPS data showed that, on average, animals occupied lower elevations, smaller areas, and fewer habitat types in winter (figure 2*a*–*c*). Across species, elevation ranges were 1598−1763 m in winter and 1634−2767 m in summer, with bison and elk tending to occupy higher elevations than smaller species in winter (figure 2*a*). Area occupied was 1.0–10.8 km^2^ in winter versus 1.3−15.4 km^2^ in summer, with bison ranging across larger areas in both seasons (figure 2*b*). Most species occupied larger areas in summer, but pronghorn occupied larger areas in winter (figure 2*b*). Habitat richness was similar across species in winter, but bison utilized the most habitat types overall (figure 2*c* and electronic supplementary material, figure S2). Smaller species used more habitats in winter than summer (pronghorn, bighorn sheep and mule deer) and vice versa for larger species (bison and elk). In both winter and summer, shrub and scrublands were the most used habitat types for most species (44.3−75.5%; electronic supplementary material, figure S2). Pronghorn rarely used evergreen forests in winter (0.7%), though this habitat was used extensively in both winter and summer by all other species (11.2−33.0%; electronic supplementary material, figure S2). All species occupied overlapping ranges but differed subtly in how they used space and habitats and, consequently, their access to food resources.

### 3.2. Seasonal variation in diet composition

**Figure 3.**
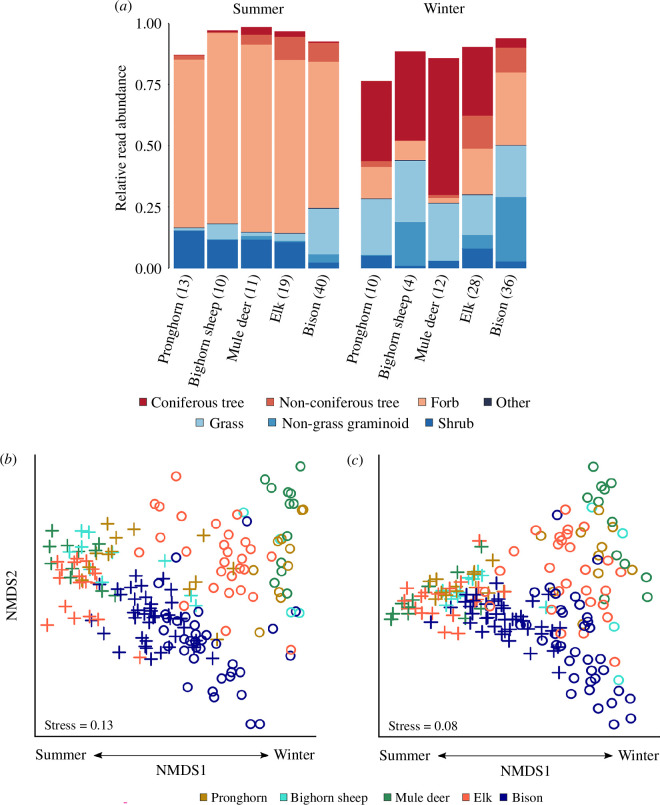
Dietary composition and niche partitioning of five large-herbivore species in Yellowstone National Park. (*a*) Stacked bars represent the proportion of each species’ diet profile that we assigned to each of seven functional groupings in summer versus winter (electronic supplementary material, appendix S3; dataset S4). At least 84% of 685 taxa in the dataset could be classified into the seven functional groups according to the USDA Plants Database (‘other’ category includes taxa that are from the family Equisetaceae and non-vascular mosses). Sample size for each species in each season are shown in parentheses. Strong dietary shifts occurred between winter (open circles, right) and summer (plus symbol, left) in (*b*) plant taxonomic composition and (*c*) functional plant type composition as visualized using NMDS and the Bray–Curtis dissimilarity metric (analyses included the ‘undetermined’ functional group). Taxonomic composition of diets differed significantly according to species (perMANOVA; pseudo-*F*_4,174_ = 9.4, *R^2^* = 0.14, *p *≤ 0.001), season (pseudo-*F*_1,174_ = 37.4, *R^2^* = 0.14, *p *≤ 0.001), and the species × season interaction (pseudo-*F*_4,174_ = 6.4, *R^2^* = 0.09, *p *≤ 0.001), as did the composition of plant functional types (species: pseudo-F_4,173_ = 11.6, *R^2^* = 0.13, *p *≤ 0.001; season: pseudo-F_1,173_ = 110.2, *R^2^* = 0.30, *p *≤ 0.001; species × season interaction: pseudo-*F*_4,173_ = 8.4, *R^2^* = 0.09, *p *≤ 0.001).

As expected, diet composition differed significantly across species (perMANOVA; pseudo-*F*_4,174_ = 9.4, *R* ^2^ = 0.14, *p *≤ 0.001) and seasons (summer versus winter: pseudo-*F*_1,174_ = 37.4, *R* ^2^ = 0.14, *p *≤ 0.001), and there was a significant species × season interaction (pseudo-*F*_4,174_ = 6.3, *R* ^2^ = 0.09, *p *≤ 0.001; figure 3*b*). Dispersion also significantly differed between seasons (summer versus winter: *F*_1,181_ = 26.6, *p *≤ 0.001) and species (*F*_4,178_ = 2.8, *p* = 0.018), with the greatest between-herd variation occurring in winter (mean sample distance to group median: 0.58). Overall, pronghorn (smallest species) had the greatest between-herd variation (0.58) while bison (largest species) had the least (0.52). However, when split by season (winter versus summer), bison had the greatest between-herd variation in winter (0.54) and the lowest between-herd variation in summer (0.42) compared to all other species (figure 3 and electronic supplementary material, tables S2 and S3, figure S5*b*–*f*). Significant dietary differences persisted when grouping dietary taxa into plant functional groups across consumer species (pseudo-*F*_4,173_ = 11.6, *R* ^2^ = 0.13, *p *≤ 0.001) and seasons (summer versus winter: pseudo-*F*_1,173_ = 110.2, *R* ^2^ = 0.30, *p *≤ 0.001), and there was a significant species × season interaction (pseudo-*F*_4,173_ = 8.4, *R* ^2^ = 0.09, *p *≤ 0.001). A key distinction between taxonomic and functional dietary differences is that the direct effect of season became much more pronounced when lumping food taxa into a smaller number of functional groups (figure 3*b,c*). Dispersion in dietary plant functional groups also differed significantly between seasons (summer versus winter: *F*_1,181_ = 110.1, *p* = 0.001) and species (*F*_4,178_ = 4.9, *p* = 0.004), with the greatest degree of between-herd variation still occurring in winter (mean sample distance to group median: 0.41). These seasonal patterns persisted when including the subset of taxa for which functional groups were undetermined (figure 3*c*; electronic supplementary material, appendix S3, figure S6).

The relative abundance of dietary plant functional groups varied markedly between summer and winter. Forbs had the greatest relative abundance in summer (40.9−70.5% per species), whereas a combination of senescent grasses (16.4−25.3%), coniferous trees (3.8–56.0%) and non-grass graminoids (0.1−26.4%) cumulatively composed most diets in winter (figure 3*a*; electronic supplementary material, dataset S4). Non-coniferous trees were most relatively abundant in winter elk diets (13.3%), while non-grass graminoids were most relatively abundant in winter bison and bighorn sheep diets (26.4 and 17.8%, respectively; electronic supplementary material, dataset S4). Bison consumed a comparatively large percentage of forbs in winter (10.1%), though the relative abundance of grasses remained similar between seasons (winter: 21.2%, summer: 18.8%; figure 3*a*; electronic supplementary material, dataset S4).

### 3.3. Hypothesis testing

**Figure 4.**
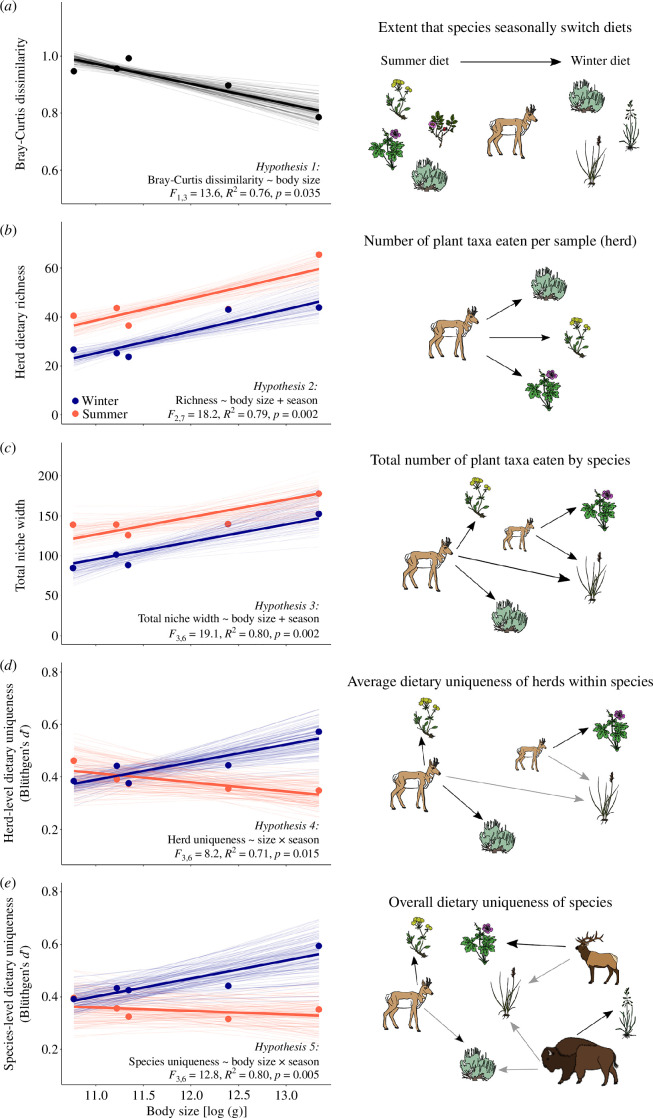
Seasonal diet changes at levels spanning herds, species and communities. (*a*) All species switched diets seasonally, but the extent of switching decreased with body size. (*b*) On average, herds of larger species consumed more taxa and all herds increased dietary richness in summer. (*c*) Total niche width was greater in summer for most species and larger species consumed more plant taxa overall. (*d*) Herd-level and (*e*) species-level dietary uniqueness changed seasonally in ways that were contingent on body size: larger species strongly increased dietary uniqueness while smaller species exhibited similar or even decreased dietary uniqueness in winter. Bray–Curtis dissimilarity and Blüthgen’s *d*′ are bounded between 0 (no turnover/specialization) to 1 (complete turnover/specialization). In all panels, thin lines indicate regressions for each of the 100 random subsamples and the thick line indicates the regression line for the best-fitting model of the mean values per species (large points).

Consistent with our hypotheses, body size was significantly correlated with seasonal dietary changes related to dietary composition, breadth and uniqueness. Our first hypothesis was that degree of seasonal diet switching (from summer to winter) would be correlated with body size, and we found a significant decline in mean Bray–Curtis dissimilarity according to size (linear regression: *β* = −0.07, *p* = 0.035, figure 4*a*). The three smallest species (pronghorn, bighorn sheep and mule deer) showed nearly complete dietary turnover between seasons (mean Bray–Curtis > 0.94), whereas bison and elk showed extensive, but more muted turnover (mean Bray–Curtis range: 0.79–0.89). Season thus had stronger effects on the diets of smaller herbivores.

Our next two hypotheses evaluated correlations between body size and dietary richness. Consistent with hypotheses 2 and 3, dietary breadth was modulated by body size and substantially greater in summer compared to winter for all species except elk (figure 3*b*,*c*). Herd-level dietary richness showed no significant body size × season interaction, but it did increase significantly with body size (*F*_1,7_ = 22.2, *p* = 0.002) and was significantly greater in summer (*F*_1,7_ = 14.2, *p* = 0.007; figure 4*b*; electronic supplementary material, tables S4 and S5). The model showed an average increase in herd-level dietary richness of approximately 14 plant taxa in summer; elk had a similar dietary richness in both seasons. Because of species-specific differences in the use of overlapping ranges (figure 2), we found that the overarching winter to summer increase in dietary richness was common across species but that species varied in the timing of peak richness (e.g. mule deer and elk peaked early in spring while others peaked later in summer; electronic supplementary material, figure S4). Similar results were found for TNW: there was no significant body size × season interaction, but TNW increased significantly with body size (*F*_1,6_ = 24.2, *p* = 0.002) and was significantly greater in summer than winter (*F*_1,6_ = 13.9, *p* = 0.007; figure 4*c*; electronic supplementary material, tables S4 and S6). The model showed an average increase in TNW of approximately 27 plant taxa in summer, though again elk showed markedly less seasonal change (figure 4*c*). When focusing on the herd-level richness of plant functional groups, there was not a significant body size × season interaction or significant effect of season, but functional richness did increase significantly with body size (*F*_1,7_ = 10.0, *p* = 0.013; electronic supplementary material, table S4, figure S3).

Our final two hypotheses considered the relationship between dietary uniqueness (*d*′) and body size. Herd-level dietary uniqueness differed among species with a significant body size × season interaction (*F*_1,6_ = 16.8, *p* = 0.006), such that it was significantly greater in winter (*F*_1,6_ = 15.1, *p* = 0.008), but there was not a significant main effect of body size (*F*_1,6_ = 3.9, *p* = 0.095; electronic supplementary material, table S4; figure 4*d*). The seasonal difference in uniqueness was fourfold greater for the two largest species than the three smaller species, which in turn showed a little change (figure 4*d*; electronic supplementary material, table S7). Similarly, the uniqueness of species-level dietary profiles showed a significant body size × season interaction (*F*_1,6_ = 11.3, *p* = 0.015) and a significant effect of season (*F*_1,6_ = 8.9, *p* = 0.024), but not a significant main effect of body size (*F*_1,6_ = 0.6, *p* = 0.471) whereby the diets of larger species exhibited markedly greater uniqueness relative to smaller species in winter (figure 4*e*; electronic supplementary material, tables S4 and S8).

### 3.4. Seasonal diets, habitat use and movement patterns

**Figure 5.**
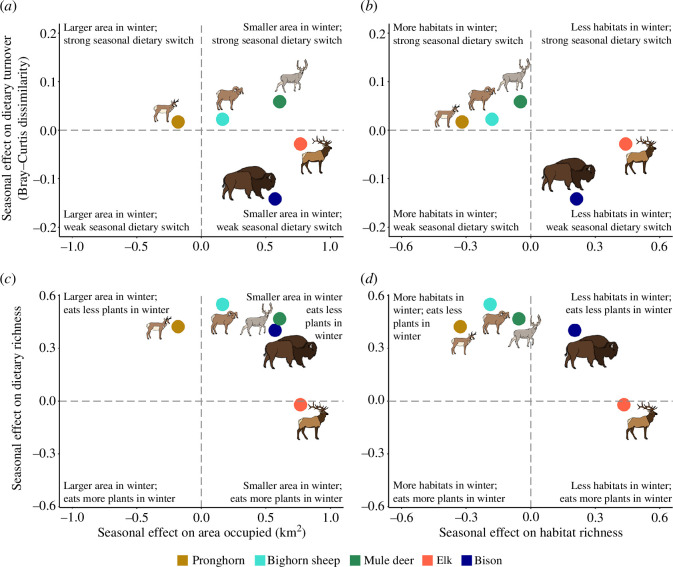
Relationship between dietary turnover (*a,b*), dietary richness (*c,d*), space use (*a,c*) and habitat use (*b,d*). Pronghorn, bighorn sheep and mule deer show relatively strong seasonal dietary switches (*a,b*) and maximized habitat richness in winter (*b,d*), but only pronghorn increased area occupied (km^2^) in winter (*a,c*). Dietary richness was maximized in summer for most species (*c,d*), but only bison and elk maximized habitat richness in summer (*b,d*).

We evaluated correlations between seasonal changes (summer versus winter) in species’ diets and how they use the landscape. Compared to the two larger species, pronghorn, bighorn sheep and mule deer used more habitat types in winter and season had a stronger effect on the degree of dietary switching, though only pronghorn occupied larger areas in winter (figure 5). Dietary dissimilarity was significantly correlated with habitat dissimilarity for the two largest species in winter, but not the three smaller species (though the test was marginally non-significant for pronghorn and mule deer and it was underpowered for bighorn sheep; electronic supplementary material, figure S7), while this correlation was significant for pronghorn, bighorn sheep and elk in summer (electronic supplementary material, figure S8). Elk was the only species to show a significant correlation between habitat and dietary dissimilarities in both seasons (electronic supplementary material, figures S7 and S8), and they generally expressed distinctive patterns in both resource and habitat use (figures 2 and 4).

## 4. Discussion

Whenever scientific frameworks provide divergent predictions about phenomena that are central to a field, it can be helpful to confront theory with data. We considered the predictions of foraging ecology and food web ecology, which differ in the mechanisms assumed to control how animals respond to seasonal variation. Analyses of dietary DNA revealed how the five predominant large herbivore species of Yellowstone National Park make use of seasonally available forage. We found strong and significant differences in diet composition and foraging behaviours that were all modulated by differences in consumer body size. Whereas all species capitalized on a regular yearly flush of high-quality herbaceous plants in summer compared to winter, there was a much more muted seasonal switch by the larger species compared to smaller species (figures 3–5). The largest species were able to maintain relatively unique diets in winter, perhaps reflecting their size-based superiority in their ability to access limited forage reserves [34,35]. In winter, larger species were likely less spatially constrained (figure 2*a,b*) because they are better able to traverse deep snow and dig for a functionally diverse suite of resources (i.e. by using their strong shoulders and larger heads [55,56]), while smaller species are likely forced to converge on dwindling supplies of higher quality resources in sheltered or wind-swept habitats [57].

The diversity and uniqueness of animal diets strongly inform the potential for competition between animals and thus have long been considered essential to our understanding of complex ecological networks [18,23,58]. Theoretical foraging ecology holds that when resources become limited and competition intensifies, dietary breadth should increase within and among species as consumers must become more accepting of less preferred foods [19] (figure 1). In contrast to this, we found that although all species added lower quality woody species to their diets in winter (figure 3*a*), the overall taxonomic diversity of their diets generally expanded in summer (figure 4*b,c*). At this fine-taxonomic level, the summertime expansion of dietary diversity contradicts predictions derived from foraging ecology and is more consistent with food web ecology (figure 1*a*). In Yellowstone, animals respond to the summer flush of high-quality, ephemeral, species-rich grassland resources [30], such as wild strawberry (*Fragaria virginiana*; electronic supplementary material, dataset S4). Despite having functionally similar diets in summer, taxonomically fine-grained niche differences persisted (figures 3 and 4), perhaps reflecting spatial variation in the diversity of forbs occurring in the habitats frequented by herbivores (figure 5 and electronic supplementary material, figure S2). For instance, in summer, fireweed (*Chamaenerion angustifolium*) was dominant in elk and mule deer diets, geranium was dominant in bighorn sheep diets, and shrubby cinquefoil (*Dasiphora fruticosa*) was dominant in pronghorn diets (electronic supplementary material, dataset S4). In contrast, in winter, species specialized on foraging for a smaller subset of resources: senescent herbs continued to be prominent in the diets of bison (e.g. Polygonaceae and Poaceae) and elk (e.g. wild strawberry and chickweed, *Cerastium arvense*), while pronghorn focused on a combination of woody vegetation (e.g. saltbush, *Atriplex* spp.) and non-native winter annuals (e.g. mustards, *Alyssum desertorum*; electronic supplementary material, dataset S4).

Despite divergent predictions about how consumers respond to seasonal resource limitations, both foraging ecology and food web ecology emphasize how body-size variation can modulate competition and niche partitioning [59–63]. We found body size to be strongly correlated with the extent of seasonal dietary switching and partitioning. Smaller species tended to adopt winter strategies consistent with OFT, as their populations converged on overlapping subsets of easily accessible resources (figures 1a, 3 and 4*d*). In contrast, the winter foraging strategies of larger species aligned better with NVH as they were less constrained to specific habitats and thus traversed habitats more individualistically to obtain heterogeneous and functionally unique diets (figures 1b, 3, 4e and 5). In summer, bison form herds that number in the thousands when they forage cohesively across massive grazing lawns in summer, but by winter they splinter into more localized groups [30,64]. In contrast, elk and the three smaller species exhibited generally stronger diet seasonality despite differences in the ways they seasonally altered their habitat and space use (figures 2 and 5), as they often form more cohesive groups in winter compared to summer [65]. Size-based differences in habitat and resource utilization were consistent with the idea that smaller species must rely on a constrained subset of higher quality and safely accessible resources, perhaps selectively feeding on and depleting these resources to levels that require larger species to utilize more abundant or less easily accessible resources of more variable qualities [33,66]. Pronghorn, for instance, moved considerably more than all other species in winter despite occupying a narrower array of habitats, likely allocating more time to searching for acceptable resources (figure 5). In light of disparities in body size and mobility, the key predictions provided by prominent ecological frameworks may not apply to all species equally.

A unifying theme in the disparate theoretical traditions that motivated our study—OFT, NVH, and food web ecology—is the central importance of allometry in shaping the animal behaviours and movement patterns that underpin complex food webs. To reconcile divergent predictions, it may help to determine whether similar, generalizable allometries of seasonal diet switching emerge in other diverse communities of large herbivores across the world. A major strength of our analysis is that it was based on a large dataset, spanning 2 years of sample collection from herds associated with GPS-collared animals, which was made possible by volunteer contributions of community scientists working with the National Park Service. However, elucidating consistent size-based differences in how species respond to changing resource availabilities will be complicated by the taxonomic, phylogenetic, and functional diversity of plant and herbivore communities across the planet. If it is true that smaller species generally face foraging constraints imposed by their need to maintain higher quality diets, whereas larger species generally require high-biomass foods that seasonally plumet in quality, then perhaps further work to integrate our knowledge of the taxonomic diversity and nutritional value of foods available to herbivores will improve our ability to accurately predict how they will respond to environmental changes [31–33]. Our comparisons of plant taxonomic diversity and functional types certainly suggest such a scenario is plausible, given the striking diversity of ways animals switched from higher quality herbs in summer to lower quality shrubs and senescent grasses in winter. Converting dietary DNA profiles into estimates of relative diet qualities in terms of metrics such as crude protein and digestible energy would allow us to evaluate this hypothesis more quantitatively [67,68].

As demonstrated in our study of Yellowstone’s large herbivores, generalist consumers can converge on functionally similar resource types yet maintain cryptic differences in the plant taxa that they select, at least when resources are abundant [20,21]. Such cryptic niche differences can complicate predictions about what it means for consumers to ‘expand’ or ‘switch’ their diets as they confront seasonal changes in resource diversity and abundance while striving to maintain nutrition and minimize niche overlap with competitors [21,36,58]. However, by utilizing high-resolution data on animal movements and diets, we revealed striking size-based associations with resource-acquisition strategies that could help reconcile the divergent predictions of OFT, NVH, and food web ecology to enable much stronger predictions about seasonal effects that span scales of biological organization from organismal foraging behaviour to the ecosystem structure.

## Data Availability

Illumina sequence data and sample metadata are available at NCBI (BioProject accession number: PRJNA780500). Bioinformatic scripts for cleaning sequence data, taxonomic assignment of sequence data, creating global and local plant reference libraries, and R code for conducting all analyses are available on Zenodo [69]. Specimen data and input FASTA files for generating the local plant reference library are available on Dryad [70]. Permanent URL for the local plant DNA barcode data is available on BOLD (releaseDS-YNPBPR2; dx.doi.org/10.5883/DS-YNPBPR2). Supplementary material is available online [71].
